# Functional Status Is Associated With Prefrontal Cortex Activation in Gait in Subacute Stroke Patients: A Functional Near-Infrared Spectroscopy Study

**DOI:** 10.3389/fneur.2020.559227

**Published:** 2020-11-05

**Authors:** Eric Hermand, Maxence Compagnat, Olivier Dupuy, Jean-Yves Salle, Jean-Christophe Daviet, Anaick Perrochon

**Affiliations:** ^1^Laboratoire EA6310 HAVAE “Handicap, Activité, Vieillissement, Autonomie, Environnement,” Université de Limoges, Limoges, France; ^2^Laboratoire EA 7369 URePSSS “Unité de Recherche Pluridisciplinaire Sport, Santé, Société”, Université du Littoral Côte d'Opale, Dunkerque, France; ^3^Médecine Physique et de Réadaptation, CHU de Limoges, Limoges, France; ^4^Laboratoire EA6314 MOVE “Mobilité Vieillissement et Exercice, ” Université de Poitiers, Poitiers, France

**Keywords:** functional near-infrared spectroscopy, dual task, gait, cognition, prefrontal cortex

## Abstract

Increasing cerebral oxygenation, more precisely the overactivation of the prefrontal cortex (PFC), reflects cortical control of gait in stroke disease. Studies about the relationship between brain activation and the functional status in stroke patients remain scarce. The aim of this study was to compare brain activation, gait parameters, and cognitive performances in single and dual tasks according to the functional status in subacute stroke patients. Twenty-one subacute stroke patients were divided in two groups according to Barthel Index (“low Barthel” and “high Barthel”) and randomly performed ordered walking, cognitive task (n-back task), and dual tasks (walking + n-back task). We assessed gait performances (speed, variability) using an electronic walkway system and cerebral oxygenation (ΔO_2_Hb) by functional near-infrared spectroscopy. Patients with better functional status (high Barthel) showed a lower PFC activation (ΔO_2_Hb) and better gait parameters in single and dual tasks compared to low-Barthel patients, who exhibited decreased gait performances despite a higher PFC activation, especially in the unaffected side (*P* < 0.001). PFC overactivation in less functional subacute stroke patients may be due to the loss of stepping automaticity. Our results underline the interest of proposing rehabilitation programs focused on walking, especially for patients with low functional capacity.

## Introduction

Stroke is associated with gait disorders, mainly characterized by a decreased gait speed (1) and a greater variability (2). Walking is further affected by challenging conditions such as simultaneous cognitive and motor tasks [e.g., dual task (DT)] (3). In stroke patients, this increased cognitive demand of walking during DT was underlined by the key role of the prefrontal cortex (PFC) (4), whose activation can be assessed by functional near-infrared spectroscopy (fNIRS) (5). Studies using fNIRS reported a greater brain activity in the PFC during DT than in single task (ST) in chronic stroke patients, implying that executive functions were primary involved in this overactivation (6, 7). Recently, we observed no difference of oxygenated hemoglobin levels (ΔO_2_Hb) between motor ST and DT in subacute stroke patients (8), highlighting a ceiling effect on brain activity observed in DT and thus the loss of stepping automaticity in these patients.

Other recent findings suggest that people with poorer mobility such as elders or neurological patients exhibits a higher PFC activation than control groups during walking, reflecting a higher cognitive demand (7, 9). In stroke, lower mobility was associated with a higher (and saturated) recruitment of the PFC in walking tasks (7, 10). Hence, the challenge for upcoming studies investigating brain activation during walking in stroke patients relies on a better understanding of the relationship between the cortical control of gait and functional independence.

The aim of this study is to compare brain activation, gait parameters, and cognitive performances in ST and DT according to the functional status in subacute stroke patients. We hypothesize that the PFC activation and the decrease in cognitive/gait performances during ST and DT are greater in stroke patients with a lower functional status.

## Participants and Methods

This was a retrospective study based on the analysis of complementary data of Hermand et al. (8).

### Participants

Twenty-one subacute stroke patients (Table 1) participated in this study, at the Limoges University Hospital. Inclusion criteria included acute or subacute stroke (<3 months), first stroke (left or right middle cerebral artery), and being able to walk 10 m. Exclusion criteria included previous neurological disease (e.g., Parkinson disease, dementia).

**Table 1:** Clinical characteristics of patients and mean values (± *SD*) of gait parameters and cognitive performance in ST and DT (mean ± *SD*).

		**LoB group**	**HiB group**	**Overall**
		**(“moderate**	**(“slight**	**(*n* = 21)**
		**dependency”)**	**dependency”)**	
		**(*n* = 8)**	**(*n* = 13)**	
**Clinical characteristics**				
Gender (Male/Female)		4/4	10/3	14/7
Age (years)		70.6 ± 10.5	66.6 ± 10.4	68.1 ± 9.4
		[57;87]	[56;86]	[56;87]
Height (cm)		167.6 ± 7.5	168.5 ± 10.2	168.1 ± 8.9
		[155;181]	[150;183]	[150;183]
Weight (kg)		71.9 ± 12.8	76.4 ± 14.8	74.7 ± 13.1
		[52;91]	[60;100]	[52;100]
Barthel Index (/100)		76.3 ± 6.4	98.1 ± 2.5^***^	89.8 ± 11.5
		[70;85]	[95;100]	[70;100]
Walking assistance (one crutch / rollator)		3 / 2	0	–
Days post-stroke		54.5 ± 39.3	68.1 ± 28.2	62.9 ± 30.9
		[11;99]	[16;93]	[11;99]
Stroke subtype (Ischemic/Hemorrhagic)		6 / 2	11 / 2	17 / 4
Affected hemisphere (Left/Right)		4 / 4	8 / 5	12 / 9
Level of education		3.6 ± 1.3	3.5 ± 1.2	3.5 ± 1.2
		[3;7]	[3;5]	[3;7]
**Gait parameters**				
*Speed (cm.s^−1^)*	ST_mot_	40.2 ± 14.8	88.5 ± 26.5^**^	71.6 ± 32.7
		[16.1;60]	[26.3;131.9]	[16.1;131.9]
	DT	32.4 ± 14.0	73.2 ± 29.6^**^	57.7 ± 31.7
		[16.1;56.5]	[21.1;133]	[16.1;133]
*Gait variability (n.u.)*	ST_mot_	18.2 ± 14.9	4.8 ± 2.5^**^	9.0 ± 10.3
		[7.5;46.8]	[1.4;10.7]	[1.4;46.8]
	DT	26.3 ± 16.4	7.1 ± 3.6^+^^**^	13.8 ± 13.5^+^
		[6.7;46.8]	[3.8;14.4]	[3.8;46.8]
**Cognitive performance**				
*% good answers*	ST_cog_	21.4 ± 11.3	28.7 ± 8.1	26.0 ± 9.8
		[11.1;44.4]	[16.7;44.4]	[11.1;44.4]
	DT	16.0 ± 8.6	16.7 ± 14.4 ^+^	16.4 ± 12.2 ^+^
		[5.6;27.8]	[0;38.9]	[0;38.9]

LoB vs. HiB:

** P < 0.01,

*** *P < 0.001. ST_cog_ vs. DT or ST_mot_ vs. DT: ^+^, P < 0.05*.

### Functional Status

The Barthel Index (BI) (11) for each patient was evaluated on test day by a trained hospital practitioner, on a 0- to 100-point scale. Patients were assigned in two groups: “slight dependency,” for a higher BI between 91 and 100 (HiB), and “moderate dependency,” for a lower BI between 61 and 90 (LoB) (12).

### Design Protocol

The patients successively performed three randomly ordered tests: a cognitive ST (ST_cog_), a walking ST (ST_mot_), and a DT. Cognitive tasks for ST_cog_ and DT followed a 2-back task (8): the experimenter, facing the patient at a distance of 1 m during ST_cog_ or walking 1 m behind him/her during DT, read aloud, and clearly a series of 20 fixed random numbers, between 0 and 10, evenly spaced in a 30-s interval. Responses were recorded with a voice recorder. The percentage of correct answers was computed for each cognitive condition, as missing or incorrect answers were accounted for as errors (8). In walking ST_mot_ and DT, patients walked through an open space at a comfortable pace for 30 s, through an 8-m GAITRite walkway (Sparta, USA), which provided speed and stride variability. One practice trial for each ST and DT task was conducted prior to experimental testing to ensure proper hearing/vision and a good understanding of each task.

### fNIRS Acquisition

Cerebral oxygenation was measured using an fNIRS system (Portalite, Artinis Medical, the Netherlands). Two optodes were placed on symmetrical prefrontal sites Fp1 and Fp2 according to the EEG 10/20 system. Acquisition was made through the Oxysoft software (version 3.0.97.1). Differential pathlength factor was set on 5 as its calculation formula does not apply to patients' age 50 years or older (13). In each condition, after a 30-s rest for baseline, patients performed the 30-s test, before a final 30-s rest phase. A 0.1-Hz low-pass filter was applied to the fNIRS signal to remove physiological and instrumental noise, and motion artifacts were corrected using MATLAB-based scripts when needed (8, 14). The relative concentrations in O_2_Hb (ΔO_2_Hb, ΔO_2_Hb-affected, and ΔO_2_Hb-unaffected in the PFC, μmol L^−1^) in the test interval (i.e., the last 20 s) were then normalized by subtracting to them the mean value of the last 10 s of baseline, immediately before the beginning of the task, that is, seated for ST_cog_ and standing for ST_mot_ and DT.

### Statistical Analysis

A Shapiro-Wilk test confirmed the non-normal distribution of the ΔO2Hb/gait/cognitive data. Friedman and Wilcoxon tests were then conducted to compare and assess the respective effects of functional status (i.e., LoB and HiB) and conditions (ST_cog_, ST_mot_, and DT) on cerebral activity (ΔO_2_Hb, ΔO_2_Hb-affected, and ΔO_2_Hb-unaffected) and gait parameters (speed, gait variability). For all analyses, the statistical significance level was set at α <0.05. Statistical analyses were performed using IBM SPSS® Statistics version 23 (IBM Corp, Armonk, NY, USA).

## Results

The LoB and HiB groups included stroke patients whose BIs range from 70 to 85 (*n* = 8) and from 95 to 100 (*n* = 13), respectively (Table 1).

### Brain Activation

**Figure 1:**
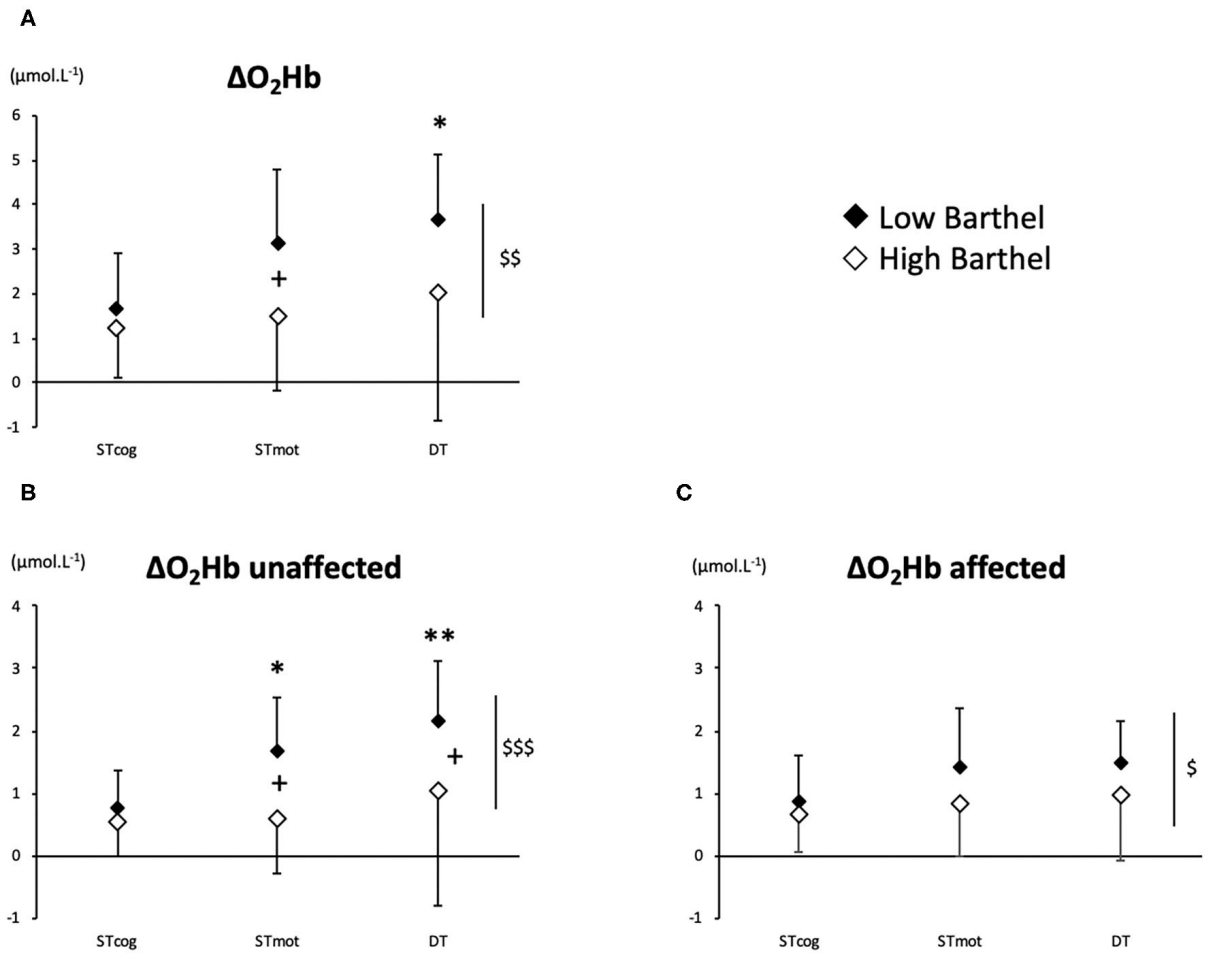
PFC oxygenation values (**A**: ΔO_2_Hb; **B**: ΔO_2_Hb-unaffected; **C**: ΔO_2_Hb-affected) for LoB (black dots) and HiB (white dots) patients in three tests: ST_cog_, ST_mot_, and DT (mean ± *SD*). LoB patients: condition vs. ST_cog_ (*P < 0.05, **P < 0.01); LoB vs. HiB (overall), ^+^*P* < 0.05; LoB vs. HiB, ^$^*P* < 0.05, ^$$^*P* < 0.01, ^$$$^*P* < 0.001.

There was an overall BI effect on ΔO_2_Hb (*P* = 0.0022), ΔO_2_Hb-unaffected (*P* = 0.0009), and ΔO_2_Hb-affected (*P* = 0.040). More precisely, LoB patients exhibited a higher activation than HiB in ST_mot_ for ΔO_2_Hb (3.13 ± 1.67 vs. 1.48 ± 1.67 μmol L^−1^, *P* = 0.025, Figure 1A) and for ΔO_2_Hb-unaffected (1.70 ± 0.85 vs. 0.63 ± 0.92 μmol L^−1^, *P* = 0.011, Figure 1B) and in DT for ΔO_2_Hb-unaffected (2.18 ± 0.93 vs. 1.06 ± 1.87 μmol L^−1^, *P* = 0.036, Figure 1B). No difference was observed for ΔO_2_Hb-affected in both LoB and HiB patients (Figure 1C).

### Gait and Cognitive Performances

An overall BI effect was observed on both speed and gait variability. More precisely, speed was higher, and gait variability was lower in HiB patients than in LoB in ST_mot_ (*P* = 0.0017 and *P* = 0.0016, respectively) and in DT (*P* = 0.0018 and *P* = 0.0013, respectively) (Table 1).

There was no BI effect on cognitive performance across all conditions.

### Effects of DT

No difference between ST and DT on PFC oxygenation was observed for the whole population (HiB and LoB patients pooled together) and for the HiB group (separately). In LoB patients, ΔO_2_Hb and ΔO_2_Hb-unaffected were lower in ST_cog_ than in DT (*P* < 0.05 and *P* < 0.01, respectively). In LoB patients, ΔO_2_Hb-unaffected was lower in ST_cog_ than in ST_mot_ (*P* = 0.028, Figure 1B).

No difference was observed between ST_mot_ and DT on gait parameters (except a trend for gait variability, *P* = 0.085) for all population. However, gait variability was higher in DT than in ST_mot_ for HiB patients only (*P* = 0.039).

There was an overall effect of DT on cognitive performances (*P* < 0.05), but this negative impact was observed only for HiB patients (*P* < 0.05) and not for LoB patients (*P* > 0.05).

## Discussion

**Figure 2:**
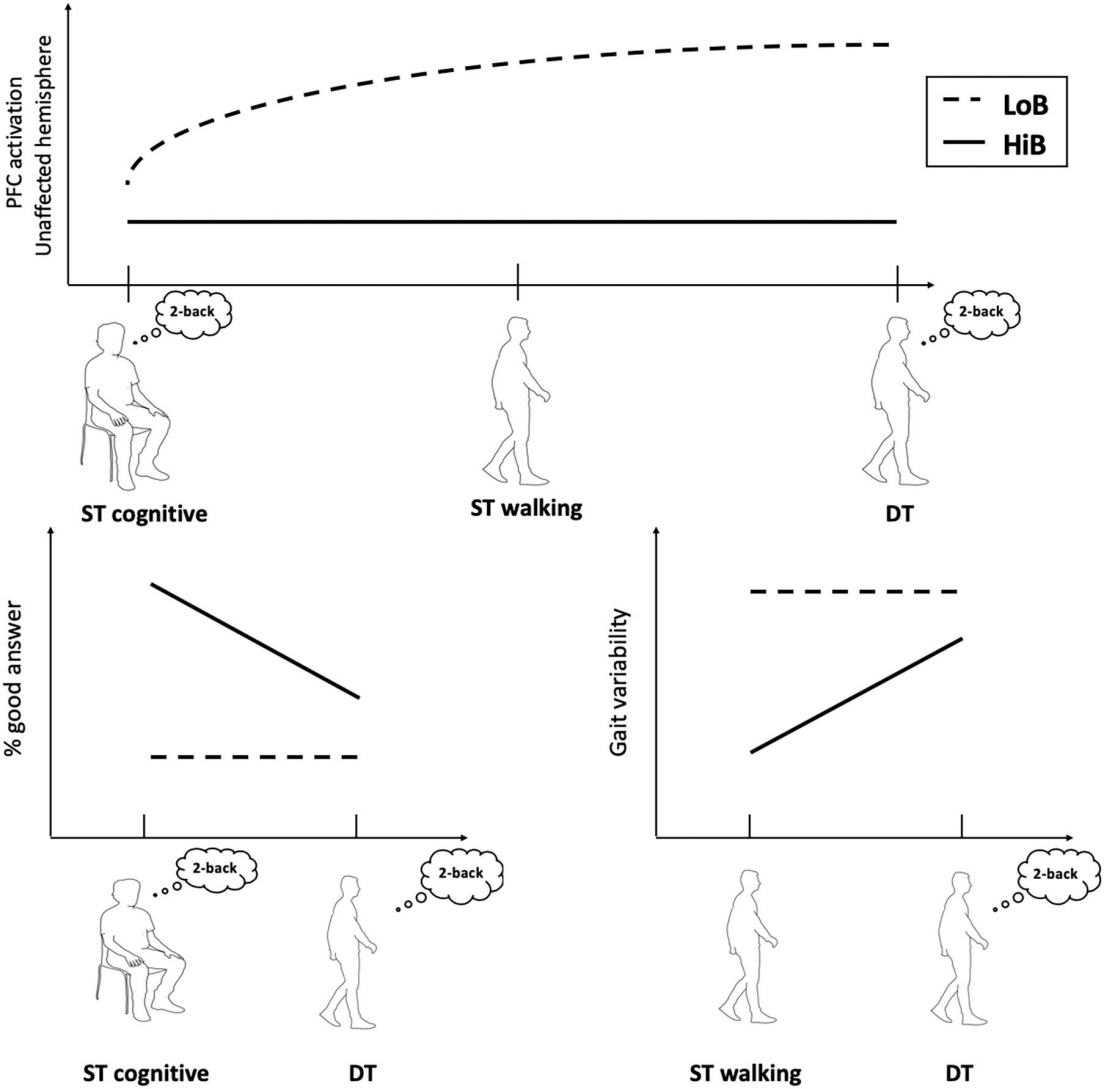
Conceptual framework illustrating cognitive–motor performances and cerebral oxygenation in walking tasks according to functional status.

First, this study shows that patients with a better functional status (HiB) showed a lower PFC activation and better gait parameters in ST and DT compared to LoB patients, who exhibited decreased gait performances despite a higher PFC activation, especially in the unaffected side (Figure 2).

LoB patients required additional attentional resources for walking, in accordance with our previous work in which ST_mot_ and cognitive–motor DT induced a PFC overactivation (vs. ST_cog_) in subacute stroke patients (8). We had highlighted the existence of a “ceiling” phenomenon in brain oxygenation induced by walking: a brain overactivation in stroke patients could be triggered by ST_mot_ and could not be further augmented by an additional cognitive load in DT (8). The present study evidences a similar phenomenon only for LoB patients, which illustrates a greater reliance on cortical control of gait in patients with poor mobility. In literature, recently published reviews on fNIRS studies during DTs in aging have interpreted the recruitment of neuronal networks in PFC as compensatory mechanisms for declines in functional efficiency (9, 15). Also, the increased PFC activity in LoB patients only seems to be associated with changes of neuronal networks caused by stroke and could be explained by models of neural inefficiency and limited capacity (16, 17).

Moreover, it is interesting to note that the functional status is associated with a sided overactivation for LoB patients during ST_mot_ and DT (vs. ST_cog_): the unaffected side was more activated (Figure 1B), as a compensatory mechanism for the affected PFC (Figure 1C), as previously observed during balance task for unaffected PFC compensating for various ipsilesional damages areas (18). Despite a higher PFC activation, LoB gait performances remained lower than HiB and confirm that a higher BI is associated with a lower gait variability (2). According to our data, we could assume that an increase of a central O_2_ availability in the unaffected PFC would not be enough to compensate for the affected PFC in LoB patients, potentially because the maximal cognitive capabilities might already be reached; as a consequence, performance in gait/cognitive tasks remain low, in ST and DT. The overactivation of the unaffected PFC could illustrate this disequilibrium between the affected and the unaffected sides, and the subsequent reassignment of cerebral tasks to the unaffected side, in whole or in part (19). We can also assume that LoB patients may exhibit a primary recruitment of unaffected PFC to compensate for the deficient side, less available to voluntary gait control. HiB patients who have better performance in gait and/or cognitive parameters would have interhemispheric activation balance in PFC, as observed in normal older subjects (7, 20). This also could involve another potential mechanism during recovery, relying on the interaction between PFC and other brain areas involved into stepping automaticity, such as premotor and primary motor cortices, which could enhance compensatory mechanisms in HiB patients, as observed in older normal subjects (21). However, in our study, we were not able to measure the activation of other brain areas, and fNIRS technology offers only a limited depth penetration that does not allow us to assess the activation of deeper cerebral structures.

Second, there was no difference of PFC oxygenation between gait conditions (ST_mot_ and DT), but we observed better gait performances (i.e., gait variability) in ST_mot_ compared to DT only for HiB. This highlights the key role of functional status on the cognitive–motor interference: unlike LoB patients, HiB patients with better recovered gait and/or cognition exhibit a decrease in their performance in DT. Compared to LoB patients in which DT does not impact the already low gait/cognitive performances, this decrease in HiB patients could be then associated to a “normal” behavior (3, 4) and hence may reflect a better recovery of walking capabilities. This discrepancy between LoB and HiB patients in subacute phase could lead to a further reflection on personalized rehabilitation modalities for stroke patients according to their functional status: LoB patients, more prone to fall risks (22), could benefit from rehabilitation strategies designed to improve stepping automaticity, whereas HiB patients may focus more on increasing the complexity of cognitive tasks.

In conclusion, our study highlights a PFC overactivation in the unaffected side for less functional stroke patients, triggered in walking conditions (ST_mot_), potentially limited by an upper limit that may not be exceeded in DT (8). This would likely be due to the loss of stepping automaticity in ST (i.e., higher-level control of gait) and then is not observed in more autonomous stroke patients. This overactivation in PFC in patients with poor mobility confirms that basic motor tasks require most of their attention resources and could be interpreted as a neural inefficiency. Also, the functional status (i.e., the Barthel Index) may represent a valuable indicator to assess both motor and cerebral recovery in stroke patients. Future studies might need to include more subacute stroke patients with various functional status, evaluated with the Fugl–Meyer Assessment (23) or by a 10-m gait test (24), and controlled sociodemographic factors. Finally, the evolution of brain activation during a follow-up of a stroke patients' cohort during rehabilitation would be interesting to investigate from acute to chronic phase of stroke.

## Data Availability Statement

The raw data supporting the conclusions of this article will be made available by the authors, without undue reservation.

## Ethics Statement

This was a retrospective study based on the analysis of complementary data of a previous study. The patients/participants provided their written informed consent to participate in this study.

## Author Contributions

AP, J-CD, and EH: study concept and design. EH: acquisition of data. EH, OD, and AP: analysis and interpretation of data. EH, OD, AP, and J-CD: drafting of the manuscript. MC and J-YS: critical revision of the manuscript for important intellectual content. All authors contributed to the article and approved the submitted version.

## Conflict of Interest

The authors declare that the research was conducted in the absence of any commercial or financial relationships that could be construed as a potential conflict of interest.
